# Revealing the alternative promoter usage of SAF/MAZ gene by bichromatic fluorescent reporter construct

**DOI:** 10.1042/BSR20171668

**Published:** 2019-01-18

**Authors:** Jianbo Ren, Dawei Guo, Xiaoyi Wang, Chao Zhang, Bo Wang, Zhe Gao

**Affiliations:** Translational Medicine Research Center, Shanxi Medical University, 56 South Xijian Nan Road, Taiyuan, Shanxi 030001, China

**Keywords:** alternative promoter, bichromatic fluorescent reporter, myc-associated zinc finger protein gene, SAF-1, SAF-3

## Abstract

The large-scale identification of putative alternative promoters study shows more than 52% of human genes are regulated by alternative promoters. The human *myc*-associated zinc finger protein (SAF/MAZ) gene have SAF-1 and SAF-3 variants transcripted from two transcription start sites (TSSs). By using SAF/MAZ promoter as a model, we set up an approach to probe how the alternative promoters are regulated in real time. We have constructed the bichromatic fluorescent reporter driven by SAF/MAZ 5′-proximal promoter plasmids from which transactivation status of SAF-1 and SAF-3 alternative promoter could be monitored by EGFP and DsRed expression respectively. The results showed that the SAF-3 expression is regulated by alternative promoters. When the bichromatic fluorescent reporter was driven by −1692/+277 or −1401/+277 SAF/MAZ promoter the dominant expression of SAF-3 would be observed in comparison with SAF-1 expression. We also identified that Elk-1 is an inhibitory transcription factor for SAF-3 expression. The temporal diversity of SAF-1 and SAF-3 expressions can be observed via bichromatic fluorescent reporters. These imply that the bichromatic fluorescent reporter driven by alternative promoter construct might be a useful tool for decoding the temporal regulatory repertoire of alternative promoter in human genes.

## Introduction

The Human Genome Project revealed that there are only 20000–25000 protein-coding genes in human genome [1]. By comparison with 6000 in yeast and 14000 in fly, the number of human genes appears too few to fit in the highly elaborated and complex system. However, high-throughput data of human genome and transcriptome identified that the phenotypic complexity of mammals is accomplished by diversity modulation mechanisms in addition to gene numbers, such as usage of alternative promoters in single gene. There are ∼50% of human genes have two or more transcription start sites (TSSs), which may be involved in gene expression regulation [2]. The use of alternative promoters would diversify transcription regulation, which is an important molecular basis for the precisely regulated complex biological systems.

*Myc*-associated zinc finger (SAF/MAZ) gene (Genbank MIM: 600999) is a CysHis2-type transcription factor widely expressed in different tissues in humans. It plays a key role in regulation of inflammation-responsive genes, cell cycle controlling genes as well as genes regulating different elements of tumor microenvironment to promote tumor growth. For instance, transcriptions of serum amyloid A (SAA), γ-fibrinogen, matrix metalloproteinase (MMP) 1 (MMP-1) and MMP14 involved in inflammation processing are regulated by SAF/MAZ protein via binding to promoters of these genes [3–5]. In non-small cell lung carcinoma cell line SAF/MAZ is involved in down-regulation of GATA4 expression, and consequently promotes tumor metastasis [6]. Also, abnormally high expression of SAF/MAZ gene triggers the progression of breast cancer and prostate cancer [7–9].

SAF/MAZ gene has multiple TSSs. A previous study on GC-rich and TATA-less promoter of SAF/MAZ gene showed that SAF-3 isoform is a better transcriptional activator and expressed during inflammation [10]. It is reasonable to speculate that the diversified expression status of SAF-1 and SAF-3 results in regulation of SAF/MAZ target gene expression to yield dynamic functional outcomes. While the previous research papers focussed on its downstream target genes [6,7,9,11], there are no details regarding the regulation of expression of the two transcription variants. Although truncation of SAF/MAZ promoter indicated that SAF-1 and SAF-3 might be driven by alternative promoter [10,12] the mechanism underlying regulation of expression of these two transcripts remains elusive.

In the present study, we constructed bichromatic fluorescent reporter [13] system driven by SAF/MAZ promoter to explore SAF-1 and SAF-3 expression regulation mechanism, instead of time-consuming RT-PCR and Western blotting. The data revealed that two transcripts are driven by different SAF/MAZ promoters. The expression SAF-1, as well as SAF-3 which are produced by upstream TSS, are down-regulated by Sp1 and Elk1. The expression status of SAF3 is diverse in different cells, suggesting that alternative promoter activation is effected by variation in levels of regulatory proteins. Our results imply that precise regulation of SAF-1 and SAF-3 promoters plays an important role for SAF/MAZ gene function in physiological and pathological procedures.

## Materials and methods

### Plasmid construction

**Figure 1:**
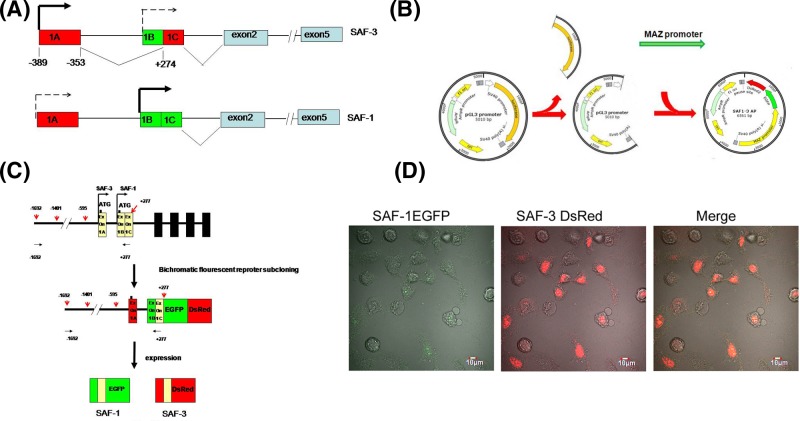
Strategy of construction of bichromatic fluorescent reporter plasmid driven by SAF/MAZ promoter (**A**) The different structure of SAF-1 and SAF-3 transcription variants is at 5′ end. The SAF-1 and SAF-3 are the different alternative splicing transcripts besides each translation from different site. (**B**) The tandem EGFP and DsRed2 ORFs and SAF/MAZ promoter at the upstream of fluorescent reporter cassettes were inserted into pGL3 plasmid instead of original Luciferase ORF to form SAF1-3 AP plasmids (the bichromatic fluorescent reporter driven by SAF/MAZ promoter). (**C**).The BamHI site at the initial codon (ATG GAT CCC) of SAF-3 was modified by BamHI digestion/blunting/re-ligation to form the different ORF from SAF-1. By this way, the EGFP and DsRed ORFs were toggled by SAF-1 and SAF-3 ORFs respectively. The black horizontal arrows stand for the PCR primer binding region for SAF/MAZ promoter subcloning. The red vertical arrows represent the truncation sites for SAF/MAZ promoter. (**D**) The SAF1-3 AP plasmids which the SAF/MAZ promoter fragment is −1401/+277 were transiently transfected into HeLa cells. The EGFP and DsRed fluorescent signals were observed under the laser confocal scanning microscopy.

The pGL3-promoter (Promega) plasmid was revised to form SAF1-3 activation promoter (SAF1-3 AP) plasmid (Figure 1B). A pair of oligos containing double Bsu36I sites, XhoI and HindIII sticky ends were inserted between XhoI and HindIII sites. The sequences of oligos are 5′-TCGAGGTGACCCTGAGGGGATCCCCTCAGGGTCGACA-3′ and 5′-AGCTTGTCGACCCTGAGGGGATCCCCTCAGGGTCACC-3′. The two sites of Bsu36I were used as restriction enzyme sites to place the SAF/MAZ 1.9-kb promoter fragment into plasmid. Another pair of oligos which consists of EcoRI, NsiI, SpeI, AflII sites and the sticky ends of XhoI and KpnI were inserted between XhoI and KpnI in pGL3 sites. By this way, the endonuclease restriction sites suitable to insert DsRed and EGFP were introduced, and SV40 promoter and polyclonal site region was removed from pGL3 simultaneously. The sequences of these two oligos are 5′-CGTCGACCTTAAGCTAGACTAGTCGGCAATGCATCCCTAGGAATTCC-3′ and 5′-TCGAGGAATTCCTAGGGATGCATTGCCGACTAGTCTAGCTTAAGGTCGACGGTAC-3′.

Then, the luciferase reporter gene was replaced by another pair of oligos between XbaI and HindIII sites. The oligo sequences are: 5′-CTAGAGCGTGACA-3′ and 5′-AGCTTGTCACGCT-3′. Three pairs of primers used to amplify human SAF/MAZ promoter, EGFP and DsRed2 were designed to amplify SAF/MAZ promoter 2.7-kb fragment, EGFP and DsRed2. The sequences of these primers are: forward 5′-CCGCCA GCTACTAACAGACC and reverse 5′-GGCCCTACTCACCTGGAATG-3′ (SAF/MAZ promoter), forward 5′-ATGCGAATTCGTGAGCAAGGGCGAGGAG-3′ and reverse 5′-TAGCATGCAT CTATTACTTGTACAGCTCGTCCAT-3′ (EGFP), and forward 5′-ATGCATGCATC CGCCTCCTCCGAGAACGTC and reverse 5′-AGTCCTTAAGCTACAGGAACAG GTGGTGGCG (DsRed2). Since the SAF-1 and SAF-3 share the same ORF, the GGATCC sequence at the beginning of SAF-3 translational starting site was modified by digesting with BamHI, blunting by T_4_ DNA polymerase and ligating by T_4_ ligase so that the EGFP or DsRed expressions were toggled by SAF-1 or SAF-3 activation, respectively (Figure 1C). For promoter deletion analysis, the SAF/MAZ promoter fragments spanning −1692 to +277 were digested by MluI, NheI, or MluI/NheI to generate different sizes of promoter fragments: the 1678 bp (−1401/+277), 872 bp (−595/+277), and 1169 bp (−1692/+277 Δ−1395/−595). The pCMV-sp1 (pN3-sp1)and pCMV-Elk1 (pCGN-Elk-1) plasmids for overexpression analysis were purchased from Addgene.

### Transfection, flow cytometry, and confocal scanning microscopy analysis

HeLa cells were cultured in DMEM medium containing high glucose (90%) supplemented with 10% FBS and plated at a density of 2.5 × 10^5^ cells per well in six-well culture plate (0712A, NEST Biotech. Co. Ltd). Transient transfections of cells were performed by Lipofectamine 3000 based on the protocol provided by Invitrogen. The transfection mixture contained 1.6 μg SAF1-3 AP plasmids, 3.2 μl Lipofectamine 3000, and 3.2 μl p3000. For co-transfection, the ratio of SAF1-3 AP plasmids and pCMV-sp1, pCMV-Elk-1, or negative control plasmids is 2.2 μg: 0.2 μg. After culturing for 48 h, the cells were washed for three times by PBS, treated by pancreatic enzymes, and filtered for flow cytometry analysis (BD FACSCalibur). For confocal scanning microscopy (Olympus FV1200) analysis, the cells were plated in four- well glass bottom culture plate (20mm 801001 NEST Biotech. Co. Ltd) specific for confocal scanning microscopy and observed fluorescent cells after 48 h.

### *In sillico* analysis and electrophoretic mobility shift assay

The prediction of transcription factors binding to SAF/MAZ promoter spanning −948 to −1 bp upstream of SAF-1 5′-UTR was performed by PROMO online software.

Nuclear extract of HeLa cells were prepared by using nuclear protein extraction kit (Beyotime, China P0027). Protein concentrations were determined by BCA assay (Beyotime, P0010S).

The Elk-1 DNA probe was designated to correspond to DNA fragments between −504 and −525 nts in SAF/MAZ promoter. The sequences of oligos used for probe were 5′-TGCTCCGCTTCCCCCACCCTCC-3′ and 5′-GGAGGGTGGGGGAAGCGGAGCA-3′. These oligos were labeled by biotin at 3′-OH terminus with Biotin-11-dUTP (Thermo Scientific R0081) catalyzed by terminal deoxynucleotidyl transferase (TdT, Beyotime Biotechnology D7093) as follows. Fifty microliters of Biotin labeling mixture containing 5 μl of oligos (1 μM), 5 μl of Biotin-11-dUTP (5 μM), 5 μl of 0.1% BSA, 10 μl of reaction buffer (5×), 0.5 μl of TdT (20 U/μl), 24.5 μl of ultrapure water was incubated in 37°C for 30 min, then at 70°C for 10 min to terminate the reaction. The labeled DNA probe was extracted by phenol/chloroform.

The binding reaction was performed in 25 μl of buffer containing 5× binding buffer (Beyotime Biotechnology) 5 μl, recombination Elk-1 protein (1 μg/μl) 12 μl, and biotin-labeled probe (0.045 pmol/μl) 8 μl. After a 10-min incubation at 25°C, the probe was added to reaction mixture and kept on incubation at same temperature for another 20 min. The products were loaded on to a 4.9% non-denaturing polyacrylamide gel in 0.5× TBE buffer. Electrophoresis was performed at 120 V for 4–6 h at 4°C. Then, the gel was equilibrated in 0.5× TBE transfer buffer for 5 min. The equilibrated gel was overlaid with a nylon membrane (RPN303B, 0.45µm, Amersham) and covered filter paper soaked with transfer buffer on both gel and membrane sides. The bands were transferred to nylon membrane semi-dried at 200–240 mA for 1 h. The probe–protein complex signals were visualized by chemiluminescence assay (Beyotime GS009) and recorded by an X-ray film.

### RT-PCR

TRIzol reagent (Invitrogen) was used for RNA extraction. The reverse reaction was performed according to PrimeScript RT Reagent Kit protocols (RR047A, Takara). The sequences of two pair of primers priming SAF-1/DsRed and SAF-3/DsRed mRNA are as following: SAF-1 forward: 5′-CCTCATGAACTCCTTCCCGC-3′, SAF-3 forward: 5′-CATCTTCCAGGGTCACC TCG-3′, and DsRed reverse 5′-CCTTCAGCTTCACGGTGTTG-3′. The qPCR was carried on in Stepone Real-Time PCR System (StepOnePlus ABI) according to the protocols of SYBR Premix ExTaqII (RR820A, Takara). For endogenous SAF-1 and SAF-3 mRNA level testing, we used the primers recommended by Ray et al. [10]. The primers for the SAF-1 cDNA PCR were 5′-CCATGTTCCCCGTGTTCCCTTGCACGCTG-3′ (forward) and 5′-GAGAACCGGGAGCAAGTCCAC-3′ (reverse). The amplification product was 271 bp. The forward and reverse primers used for SAF-3 mRNA level testing were 5′-CCGCCATGGATCCCAGCAACTGGAGCAGC-3′ and 5′-GAGAACCGGGAGCAAGTCCA C-3′, respectively. The size of primer amplicon was 208 bp. Primers for GAPDH are 5′-TGCACCACCAACTGCTTAG-3′ (forward) and 5′-AGAGGCAGGGATGATGTTC-3′ (reverse). The size of PCR product is 177 bp.

### Live cell imaging

HeLa cells were transfected with SAF1-3 AP plasmid. After 27 h, the cells expressing bichromatic EGFP or DsRed were imaged for 48 h. The fluorescent signals in MKN-45 cells were detectable at 41–57 h post-transfection. Wide field epifluorescence live-cell imaging (DeltaVision, Applied Precision Inc.) analysis was performed in an environmentally controlled chamber (37°C, 5% CO_2_), capturing images at 12-min intervals. Data were collected from background-corrected time-lapse movies in ImageJ software.

### Statistical analysis

SPSS17.0 was used for data analysis. Comparison between two groups were performed using ANOVA analysis. Significance was defined as *P*<0.05.

## Results

### Constructing bichromatic fluorescent report to probe the alternative promoter usage on SAF/MAZ gene

SAF-1 and SAF-3 transcript variants are different at 5′ ends, thus each protein differs in N′ terminal region. Comparison of N′ terminal region of SAF-3 and SAF-1 indicates that the SAF-3 initial translation sites is upstream of SAF-1 and SAF-3 is produced by alternative splicing (Figure 1A). Our aim was to construct a bichromatic fluorescent reporter driven by SAF/MAZ promoter that would reflect SAF-1 and SAF-3 expression separately. Naturally, the SAF-1 and SAF-3 share the same ORF. We modified the SAF-3 reading frame by using restriction enzyme cut, blunting and ligation strategy to fit for bichromatic fluorescent reporter so that SAF1 and SAF-3 ORFs located upstream of EGFP-DsRed could be reported by one of two fluorescent proteins (Figure 1B,C). The HeLa cells transfected with SAF1-3 AP plasmids were observed under the confocal laser scanning microscopy. We observed that red fluorescence representing SAF-3 variant was much stronger than that of green one representing SAF-1 (Figure 1D).

### Distinguishing SAF-1 and SAF-3 expressions by different promoters of SAF/MAZ gene

Previous studies by Ray et al. [10] suggested that SAF-3 transcript may be driven by promoter located upstream of SAF-1 promoter by using cat reporter assay, by which SAF-1 and SAF-3 share the same ORF. To clarify the different genomic regions that confer SAF-1 and SAF-3 expression separately we then generated five SAF/MAZ promoter constructs, −1692/+277, −1401/+277, −595/+277, −1395/−595, and −1692/+277Δ−1395/−595. These SAF/MAZ promoter activations were examined by flow cytometry assay. Activities of each promoter deletion fragment was measured four times to obtain statistically meaningful data.

The activities of these promoter were considered as an average of fluorescence intensity (FI) amongst over 40000 cells. For SAF-3 expression, the construct that showed high promoter activity were −1692/+277 and 1401/+277 fragments. Removal of 806 upstream of this region led to sharp decrease in promoter activity (−595/+277 and −1692/+277Δ−1395/−595).

**Figure 2:**
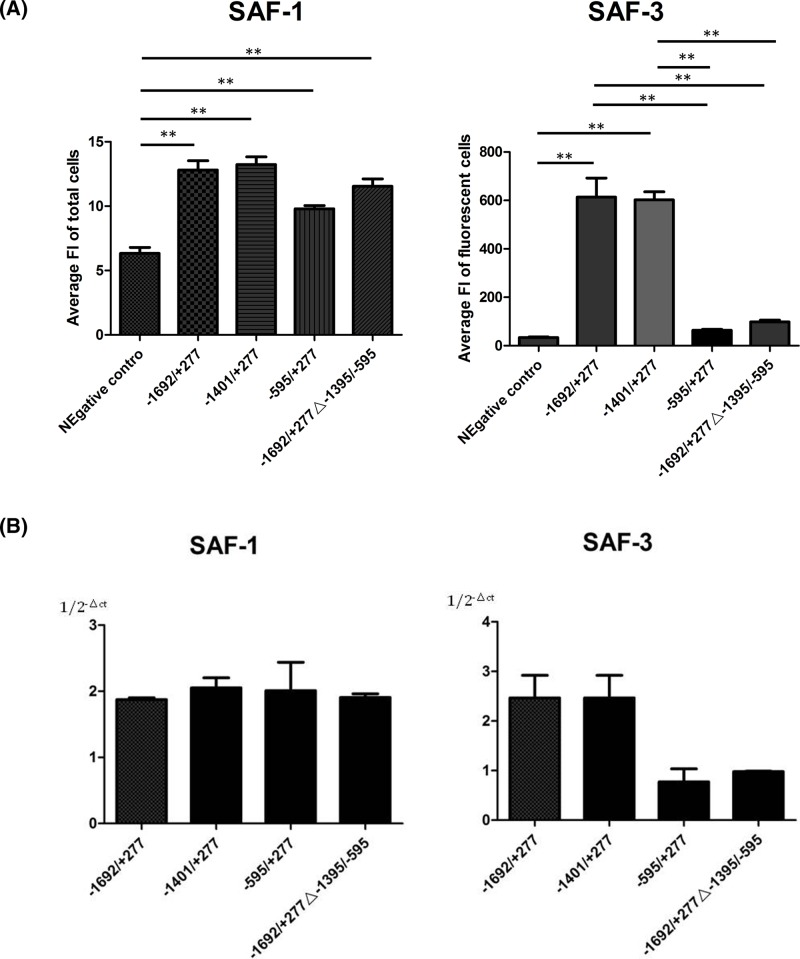
Expression of SAF-1 and SAF-3 variants driven by alternative promoters The biochromatic fluorescent reporters were driven by −1692/+277 promoter of SAF/MAZ gene or −1401/−277, −595/+277, and −1692/+277Δ−1401/−595 SAF-1. The average FIs of cells were analyzed for SAF-1 and SAF-3 by flow cytometry (**A**).The data shown represent the difference of mean ±SEM of three separate expreiments between two groups indicated by line below star symbols (**P<0.01). The SAF-1 or SAF-3 mRNA levels were measured by qPCR using the primers specific to SAF-1 and SAF-3 in bichromatic fluorescence plasmids (**B**).

Although there were statistically meaningful differences amongst these four constructs for SAF-1 expression by flow cytometry analysis, the change of SAF-1 expression was not as obvious as for SAF-3 expression (Figure 2A) .

These results indicate that SAF-1 and SAF-3 expressions were regulated by two distinct promoters.

To test each fluorescent reporter expressed from SAF-1 and SAF-3 ORF, we performed RT-PCR using primers specific for SAF-3/DsRed and SAF-1/DsRed mRNA. The ratio of SAF-3/SAF-1 real-time PCR signal was consistent with flow cytometry assays (Figure 2B).

### Identifying a new transcription factor involved in regulation of SAF-3 expression and probing the endogenous SAF-1 and SAF-3 expression status in HeLa cell

**Figure 3:**
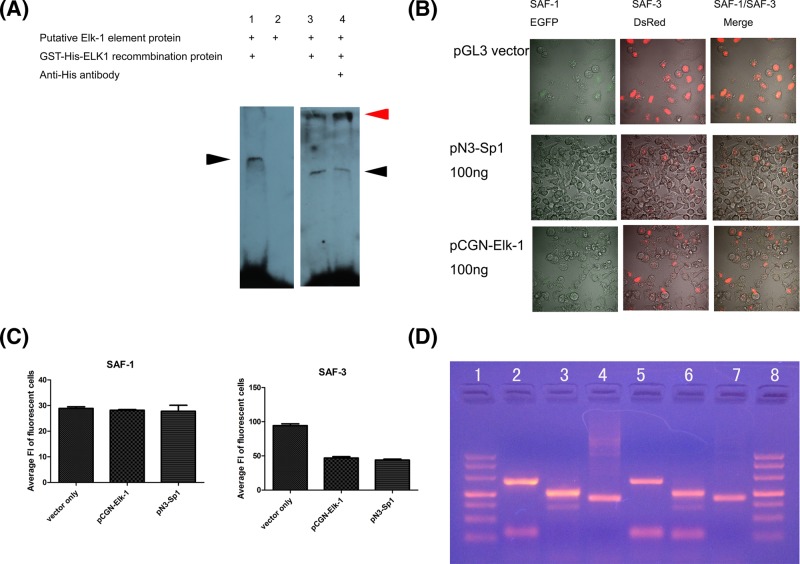
Repression of SAF-1 and SAF-3 promoter by transcription factor Elk-1 and endogenous SAF-1/SAF-3 expression Elk-1 *cis*-element on SAF/MAZ promoter was identified by EMSA (**A**). Horizontal black and red arrows represent the specific protein/DNA binding bands and anti-His/His tagged ELK-1/DNA probe supershift bands, respectively. The bichromatic fluorescent reporter plasmids were transiently co-transfected with empty plasmid, pCGN-Elk-1 and pN3-Sp1 into HeLa cells. The SAF-1 and SAF-3 promoter activation status were tested by either laser co-focus microscopy (**B**) or by flow cytometry analysis (**C**). The endogenous SAF-1 and SAF-3 mRNA expression status in HeLa cells are shown by (**D**). In (D), lanes 1 and 8 are DNA markers, lanes 2 and 5 are SAF-1 mRNA levels (271 bp), lanes 3 and 6 are SAF-3 mRNA levels (208 bp), and lanes 4 and 7 are GAPDH mRNA levels (177 bp). Abbreviation: EMSA, electrophoretic mobility shift assay.

We next asked whether SAF-1/SAF-3 expressions were regulated by other transcription factors besides Sp1 and SAF/MAZ. We then theoretically searched the transcription binding sites in SAF/MAZ promoter located −948/−1 by using PROMO bioinformatics tool. An ETS-domain containing transcription factors, Elk1 *cis*-element positioned at nucleotides −521/−517 has been predicted [14,15]. The gel shift assays were performed to confirm the binding of ELK1 recombination protein at this site. Protein interacting with ELK1-binding site was observed using the biotin-labeled ELK1 oligos. His-tagged ELK1 DNA binding activities were supershifted by anti-His antibody (Figure 3A).

Song et al. reported [12] that expression of SAF-1 were repressed by Sp1 and SAF/MAZ. The *cis*-elements of Sp1 and SAF-1 are mainly located at nucleotides −599 to +1, upstream of SAF-1 starting transcription site. The plasmids expressing Sp1 were co-transfected with SAF1-3 AP plasmids into HeLa cells. As expected, SAF-3 promoter was inhibited when Sp1 was overexpression (Figure 3B,C). We then focussed on the effects of Elk1 in transactivation of SAF-3 alternative promoter. The SAF1-3 AP plasmids were used to transfect HeLa cells in presence or absence of Elk1 overexpression plasmid. Both the SAF-3 and SAF-1 variants’ expressions were inhibited in the presence of ectopically expressed Elk1 (Figure 3B,C).

Together these observations indicate that the Sp1, SAF/MAZ as well as Elk1 are repressive transcription factors for exogenous SAF-3 variant.

In order to know to what extent of relation between endogenous SAF-1 and SAF-3 expressions and expression status from bichromatic fluorescent reporter. We tested the endogenous SAF-1 and SAF-3 mRNA in HeLa cell by using specific SAF-1 and SAF-3 primers recommended by Ray et al. [10]. The result showed that our bichromatic fluorescent reporter SAF-1 and SAF-3 expressions did not fully match the endogenous SAF-1 and SAF-3 expressions in HeLa cells (Figures 1D and 3D).

### Observing the SAF-1 and SAF-3 expression under live cell imaging system

**Figure 4:**
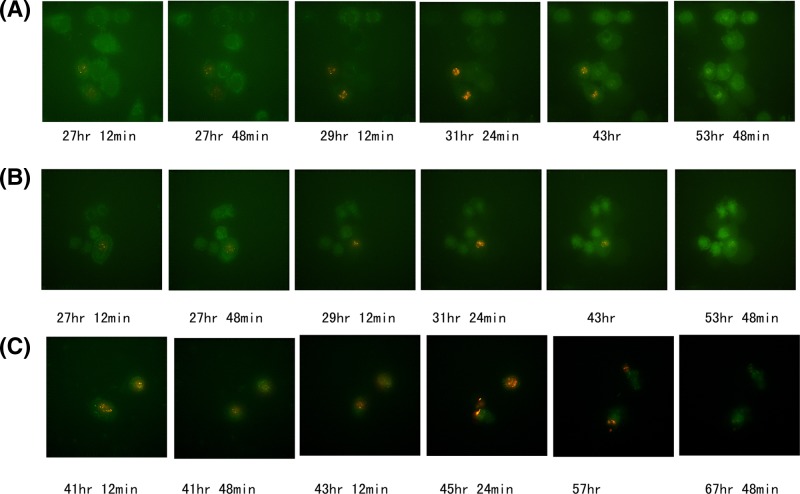
Two different activation patterns of SAF-1 and SAF-3 promoters The bichromatic fluorescenct reporter plasmids were transiently transfected into HeLa cells (**A**,**B**) and Supplementary Movies SA and SB) and MKN-45 cells ((**C**) and Supplementary Movie SC). The SAF-3 promoter activation lasted ∼16 h (27 h 48 min to 43 h in HeLa cells and 41 h 48 min to 57 h in MKN-45), while the weak SAF-1 promoter activation were from 27 h to 53 h 48 min in HeLa cells. In MKN-45 cells the SAF-1 promoter was much weaker than that of HeLa cells ((C) and Supplementary Movie SC).

Previous study shows that SAF-1 is widely involved in arthritis and cancer processing [4–7,9] and SAF-3 may be an important fine-tuning regulation factor for SAF-regulated genes as a much higher transactivation potential variant compared with SAF-1. We examined the temporal order of SAF-1 and SAF-3 promoter activation under the live cell imaging system. The results showed that some but not all HeLa cells expressed SAF-3-DsRed which appeared red after 27 h of transfection and lasted ∼16 h (27–33 h after transfection). SAF-1-EGFP signal can be seen all the time albeit the signal was much weaker than that of SAF-3-DsRed’s (Figure 4 and Supplementary Movies SA and SB).

For gastric carcinoma cell line MKN-45, the SAF-3-DsRed appeared after 41 h transfection and lasted ∼16 h. Unlike the HeLa cell SAF-1 signal was rarely observed (Figure 4 and Supplementary Movie SC).

This finding implies that the regulation of SAF-1 variant expression is tissue specific.

## Discussion

For human genes, about half of genes have multiple TSSs. Some of them are regulated by alternative promoters [16–18]. Identification of alternative promoters has been accomplished mainly by biochemical approaches such as RT-PCR and Western blotting [19,20]. Presently, split fluorescent protein ORFs, in which the read out for alternative splicing by the formation of a full length of one triggered by alternative intron exclusion, have been reported as useful [21]. The fluorescence reporter protein have benefit to study alternative splicing in real time. Furthermore, Orengo et al. [13] designed bichromatic fluorescent reporter to test the relative quantities of two alternative splices, on which the green and red fluorescence expression were toggled by the two different splice cassettes at the upstream of dsRED and EGFP, respectively. Therefore, the ratio of two splicer expression has been shown in single cell at real time. This prompts us to probe the SAF-1 and SAF-3 transcription dynamically driven by SAF/MAZ promoter using bichromatic fluorescence reporter system. Naturally, SAF-1 share the same ORF with SAF-3. We inserted 4 nts in SAF-3 coding region to make different ORFs between SAF-1 and SAF-3 in order to switch on EGFP or dsRED when SAF-1 or SAF-3 are expressed, respectively. Our design differs from earlier SAF/MAZ promoter activation experiments in which two variants were indistinguishable because of same ORF for SAF-1 and SAF-3 in genomic SAF/MAZ gene [10]. So, our present data show the SAF-1 and SAF-3 expression status separately. The approach paves the way to investigate mechanisms of activation of alternative promoters in SAF/MAZ gene.

To rule out the fluorescence protein that were not transcribed from SAF-1 or SAF-3 promoter, the RT-PCRs were performed using primers specific to SAF/MAZ promoter fluorescence construct. Our experiments showed that both SAF-3 and SAF-1 mRNA levels are consistent with DsRed and EGFP intensities by both flow cytometry and fluorescence microscopy assay.

Observations of fluorescence indicated that SAF-3 was dominant expressing variant driven by −1692/+277 and −1401/+277 SAF/MAZ promoters when the constructs were transfected into HeLa cells within 40 h. The SAF-1 fluorescence signals in −1692/+277 and −1401/+277 constructs were much weaker than SAF-3. The SAF-3 fluorescence were lost on the 5′ end shortened −594/+277 construct. Of note the SAF-3 but not SAF-1 fluorescent signal observed for the −1692/+277 was also lost when ∼800 bp of −1395/−595 were deleted (−1692/+277 Δ−1395/−595). Therefore, we conclude that there at least two distinct promoters regulating SAF-1 and SAF-3 variant expression, respectively. The results also suggest that the regions between −1395 and −595 may contain area for binding positive *trans*-acting regulatory elements for SAF-3 expression.

To search for new *trans*-acting regulating elements on SAF/MAZ promoter, we then performed the *cis/trans* factor prediction within the SAF/MAZ promoter region spanning −948 to −1 bp using PROMO3.0 online software under the 9% maximum matrix dissimilarity rate condition. Although there were multiple binding sites for some of transcription factors *in silico* analysis, such as pax-5, p53, C/EBPβ, ETF, GR-α, GCF, E2F-1STAT4, and RXR-α a putative ELK1 *cis*-element at −518/−510 which is near the silencer element containing region was identified by electrophoretic mobility shift assay (EMSA) unexpectedly (Figure 3A). The silencer element containing region reported by Song et al. also consisted of negative regulation of the SAF/MAZ expression elements binding by Sp1 and SAF/MAZ [11].

In an attempt to identify if SAF-3 transcription was effected by Elk-1, the plasmids on which Sp1 and Elk-1 were constitutive expressions were co-transfected with bichromatic fluorescence reporter plasmid into HeLa cells. We observed that the SAF-3 promoter activities were markedly decreased on condition of Sp1 or Elk-1 overexpression compared with SAF-1 promoters. It suggests that Sp1, SAF/MAZ as well as Elk-1 are down-regulated transcription factors for SAF-1 and SAF-3 promoters [11].

We do not know yet the detailed significance why ELK-1 is involved in regulation of transcription of SAF/MAZ variants from alternative promoter. However, a pile of data show that both SAF/MAZ and Elk-1 play central role in inflammation and cancer pathogenesis. There are some reports that MMP-1, MMP-9 [22], and MMP-14 genes can be induced by SAF-1 as the transcription factor [5], while p-Elk-1 binds to MMP-9 promoter and thereby induces transcription of MMP-9 in astrocytes [23]. Therefore, the events imply both transcription factors of SAF-1 and Elk-1 are involved in the processes of breakdown of extracellular matrix (ECM), which play a key role for cell reproduction, tissue repairing, and tissue remodeling during the embryonic development or inflammation processing [24].

Also, under cytokine activation conditions, binding of IL-1 to its cognate receptor results in Elk-1 activation [25] and SAF-1 can be activated and binds to human γ-fibrinogen gene promoter after IL-6 is stimulated in human liver-derived HepG2 cell line [3].

The data above suggested that taking part in MMPs and cytokine activation processes is one of various indirect collaborative manners for SAF-1 and Elk1. In our present study, we provide the evidence of down-regulation of SAF-3 and SAF-1 expression by Elk1. Hypothetically, by means of these transcription factors ‘cross-talking’ directly the alternative promoter transcripts of SAF/MAZ gene can be precisely regulated.

Alternative promoter usage, which underpins the formation of transcripts differing in their first exon is one of the mechanisms involved in cause of diversity transcriptomes [26–28]. The data from 200 primary cell types and over 400 cell lines in human reveal that there are at least four TSSs for every gene on an average [29]. A lot of evidence shows that expressions from alternative TSSs are often regulated in a spatio-temporal manner [26,30], such as dopamine receptor gene [31] and dopamine transporter (SLC6A3) [32]. With the aid of bichromatic fluorescent report plasmids, we observed cell-to-cell variable SAF-3 fluorescence signals under live cell imaging system. That is SAF-3 was expressed in some cells and others expressed both SAF-1 and SAF-3. The cell-to-cell variability for SAF-3 promoter activation is likely due, at least in part, to variation in levels of SAF-3 promoter regulatory proteins.

An interesting phenomenon was that the SAF-1 promoter was inhibited in these cells while SAF-3 promoter was activated. The inhibition of SAF-1 promoter lasted until the SAF-3 fluorescent signals were extinct. Although the mechanism of mutual inhibition of SAF-1 and SAF-3 promoters remains elusive, we speculate that the mutual inhibition of alternative promoter is needed for fine-tuning the function of SAF/MAZ gene.

Based on our observation, SAF-1 promoter appears at constant but weak activation status in bichromatic fluorescent reporter system while SAF-3 promoter activation shows the transient and burst pattern. This implies that the two kinds of promoter activation pattern are conducive to co-operation of these two variants so as to precisely regulate their target gene transcription.

There are weaknesses for our bichromatic fluorescent reporter driven by alternative SAF/MAZ promoter system. We observed endogenous SAF-1 and SAF-3 mRNA expression status by RT-PCR in HeLa cells and the difference between SAF-3 and SAF-1 mRNA level were not distinct, although SAF-3 level was slightly higher than SAF-1 (Figure 3D). The results were inconsistent with bichromatic fluorescence observation (Figure 2A).

We then observed the endogenous SAF-1 and SAF-3 mRNA under perturbation of Sp1 and ELK-1 expression condition. The alteration of endogenous SAF-1 and SAF-3 mRNA expression are consistent with the change in the alternative SAF/MAZ promoter system by overexpression or knocking down these two *trans*-acting factors (data not shown).

The results above shows that our bichromatic fluorescence SAF-1 and SAF-3 expression did not fully reveal the both endogenous transcripts expression status. It hints that there may be more DNA elements for regulation of SAF-1 and SAF-3 transcriptions other than −1692 to +277 MAZ promoter fragment. The regulation mechanisms of SAF-1 and SAF-3 promoter activation under endogenous SAF/MAZ promoter need to be further analyzed [33].

## Supporting information

**Supplementary Video S1:** 

**Supplementary Video S2:** 

**Supplementary Video S3:** 

## Abbreviations

DMEM: Dulbecco’s Modified Eagle’s Medium
DsRed: *Discosoma sp.* red fluorescent protein
EGFP: enhanced green fluorescent protein
Elk-1: Ets-like transcription factor
ETS domain: E twenty-six domain
GAPDH: Glyceraldehyde-3-phosphate dehydrogenase
GATA-4: GATA binding protein 4
IL-1: Interleukin-1
MMP: matrix metalloproteinase
QPCR: Quantitative Polymerase Chain Reaction
RT-PCR: Reverse Transcription Polymerase Chain Reaction
SAF1-3 AP: SAF1-3 activation promoter
SEM: standard error of the mean
TdT: terminal deoxynucleotidyl transferase
TSS: transcription start site

## Appendices

Supplementary Movies SA, SB, and SC.
